# Multi-Scale Modeling in Morphogenesis: A Critical Analysis of the Cellular Potts Model

**DOI:** 10.1371/journal.pone.0042852

**Published:** 2012-09-11

**Authors:** Anja Voss-Böhme

**Affiliations:** Center for Information Services and High Performance Computing, Technical University Dresden, Dresden, Germany; University of Zurich, Switzerland

## Abstract

Cellular Potts models (CPMs) are used as a modeling framework to elucidate mechanisms of biological development. They allow a spatial resolution below the cellular scale and are applied particularly when problems are studied where multiple spatial and temporal scales are involved. Despite the increasing usage of CPMs in theoretical biology, this model class has received little attention from mathematical theory. To narrow this gap, the CPMs are subjected to a theoretical study here. It is asked to which extent the updating rules establish an appropriate dynamical model of intercellular interactions and what the principal behavior at different time scales characterizes. It is shown that the longtime behavior of a CPM is degenerate in the sense that the cells consecutively die out, independent of the specific interdependence structure that characterizes the model. While CPMs are naturally defined on finite, spatially bounded lattices, possible extensions to spatially unbounded systems are explored to assess to which extent spatio-temporal limit procedures can be applied to describe the emergent behavior at the tissue scale. To elucidate the mechanistic structure of CPMs, the model class is integrated into a general multiscale framework. It is shown that the central role of the surface fluctuations, which subsume several cellular and intercellular factors, entails substantial limitations for a CPM's exploitation both as a mechanistic and as a phenomenological model.

## Introduction

### Motivation

Understanding the mechanisms that control tissue organization during development belongs to the most fundamental goals in developmental biology. Quantitative approaches and mathematical models are essential to deduce the consequences of existing morphogenetic hypotheses, thus providing the basis for experimental testing and theoretical understanding. One approach to questions concerning patterning in developing organisms is to consider tissues as huge populations of cells which behave according to certain rules that depend on their genetic programs and inner structure as well as on environmental influences. To a large extent, the environmental influences are constituted of the states and actions of directly neighboring cells. Then, tissue organization can be understood as emergent behavior that results from local intercellular interaction, being the result of processes at different spatio-temporal scales. To understand the relevance of particular factors on the subcellular or cellular scale for tissue organization, the development and analysis of suitable mathematical models is indispensable.

Physics has a long history in modeling and analyzing problems where multiple spatio-temporal scales are involved, so-called *multi-scale* problems. Therefore, existing frameworks often originate in statistical physics. For instance, so-called *equilibrium models* are designed to study the macroscopic-scale characteristics of many particles which interact on a microscopic scale at equilibrium, that is when the temporal evolution has relaxed to a stationary state. Often these models are analyzed with the help of Markov chain Monte Carlo methods, for instance the Metropolis algorithm [1]. In these cases, an auxiliary dynamics is constructed which drives the system from an arbitrary initial state towards the equilibrium state that shall be studied.

One attempt to tackle patterning processes in development has been to adopt a suitable equilibrium model of statistical physics together with an auxiliary dynamics and modify it such that the needs of developmental biology are met. This approach was pursued by Glazier and Graner in a series of papers such as [2]–[7]. They took a model which was originally developed in solid state physics to study ferromagnetism. Adapting the term which describes the interdependence structure of the individual units at the lower spatial scale and modifying the updating algorithm of the Metropolis algorithm, they obtained a dynamical system that mimics observed biological behavior seemingly realistically. The thus proposed model has been called *cellular Potts model (CPM)* or *Glazier-Graner-Hogeweg model*. It was first used in computational biology for a theoretical study of cell sorting, a phenomenon where an initially mixed cell population segregates into homotypic clusters presumably due to type-specific differences in the strength of intercellular adhesion. Subsequently, the model has been extended more and more to address a variety of biological questions in different contexts including tumor formation and progression, see for instance [5], [8]–[15]. In general, CPM-based models are used to simulate the collective behavior of interacting cells and to predict the emergent behavior at the tissue scale.

In a CPM, biological cells are described as spatially extended but internally structureless objects that cover several nodes of a regular lattice. Cells move or change their shape by annexing or rejecting single nodes according to a rule which is dependent on a pre-specified cellular and subcellular interdependence structure. The resulting cell behavior in a CPM visually resembles membrane fluctuations and pseudopod protrusions as observed for biological cells. Due to the cells' subdivision into subcellular parts, a CPM is capable to model cells with type-specific sizes and morphologies.

There are only a few model classes besides the CPM that allow to study interacting cell populations with non-isotropic and type-specific cell morphologies. Established models with a similar spatial resolution are the Vertex Model [16]–[18] and the Subcellular Element Model [19], [20]. They are spatially continuous models of different origin and nature than the CPM. Their specific advantages and drawbacks render them in some respects comparable to the CPM, however a detailed analysis of the similarities and differences is left to further study. In this paper, the focus is laid solely on the properties of the CPM.

Notice that, despite its popularity in theoretical biology, the modeling framework of cellular Potts models has received little attention yet from mathematical and modeling theory. Though the CPM has its origin as an equilibrium model – for which the mathematical properties are well-understood – , the theoretical fundament of the CPM framework and area of sound application need further clarification. This is because there has been a paradigm shift when devising the CPM for patterning processes in developmental biology. CPMs are utilized as *kinetic models*, that is to study or identify major dynamical determinants of a temporarily evolving process. However, being no longer equilibrium models, they are not automatically appropriate kinetic models. To distinguish the problems where CPMs can be effectively applied as kinetic models for tissue organization and to assess the mathematical properties of this model type, the modeling framework of cellular Potts models is subjected to a theoretical study in this paper. It is explored under which conditions a CPM is an appropriate dynamical model for intracellular interaction and what its principal behavior at different time scales characterizes.

It is shown that the modifications in the Metropolis algorithm have a dramatic impact on the long-time behavior of the model. In the long run, the cells consecutively die out, independent of the specific interdependence structure that characterizes the model. At smaller time scales, when spatial correlations have already established but the stationary state is not yet reached, the model outcome is the result of an interplay between behavior that is controlled by the modeler via the specification of the cellular interdependence structure and an additional, hardly controllable impairment that is due to the modification of the Metropolis updating scheme. Dwelling deeper into the question to which extent the model can be exploited to derive reliable predictions of the macroscopic behavior that can be expected from particular microscopic interactions, the CPM is integrated into a general multiscale modeling framework. It is argued, that the CPM's resolution below cell level allows to overcome the lattice anisotropy and to model cells with flexible and adaptive morphologies. However, the characteristic to model intercellular interaction exclusively via surface fluctuations entails substantial constraints with respect to the level of detail from the subcellular scale that can be traced by the model. In addition, the cells' subdivision into subcellular parts necessitates non-local interaction rules to control the cellular morphology. These rather technical terms hinder the application of some powerful mathematical methods, such as rigorous spatio-temporal limit procedures, for the analysis of the emergent macroscopic behavior. Thus, the flexibility in the cells' morphologies comes at the price of less control over the model's cellular behavior and intercellular interaction and of limited analytic tractability, both leading to a reduced mechanistic understanding. It is clear from many successful applications of CPMs to deep biological questions, e.g. [4], [5], [9], [21], that the CPM frameworks is an expedient modeling approach if cell size, cell shape, or cell polarity essentially affect the intercellular interaction rules and, in particular, if the cellular morphology is considered adaptive to the surrounding cellular environment. However, to value the contribution of a model to the underlying biological problem, it is essential to understand the model's theoretical basis and construction and to discuss openly its power and limitations. There exists considerable empirical knowledge of how CPMs behave for certain choices of the parameter values which has been obtained from extensive CPM simulations. Nevertheless, it is necessary to complement empirical experience with rigorous analytical arguments to provide more clarity about the structural properties of CPMs and to distinguish reliable facts from mere beliefs. This also helps to expose existing inconsistencies and drawbacks of the model class as a basis and encouragement for further discussions and developments. This paper shall be a contribution towards this objective.

### Mathematical model description

**Figure 1:**
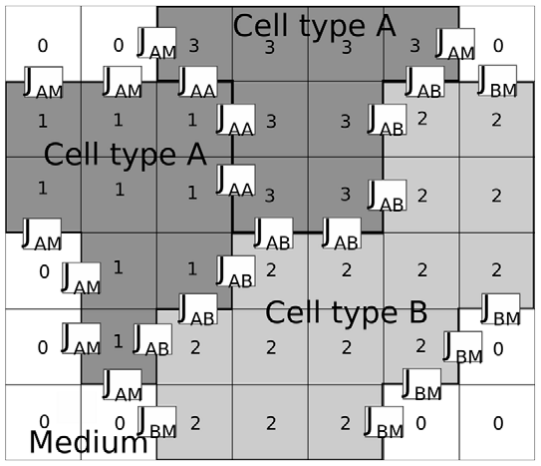
Cell-surface interaction in the cellular Potts model is regulated by the surface energy coefficients. Three cells with cell indices 1, 2 and 3, respectively, each one covering several lattice sites, interact with each other at the cell surfaces. The cells 1 and 3 are of type A, depicted in dark grey, the cell 3 is of type B, depicted in light grey. The strength 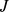 of the interaction depends on the cell types. There are also interactions between the cells and the medium (white, cell index 0). Possible boundary interactions are not shown.



A CPM assigns a value 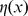 from a set 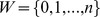 to each site 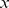 of a countable set 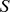, cp. Fig. 1. The set 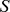 resembles the discretized space and is usually chosen as a two- or three-dimensional regular lattice. The set 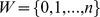 contains so-called *cell indices*, where 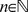 is the absolute number of cells that are considered in the model. The state of the system as a whole is described by *configurations* 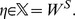 Given a configuration 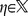, a *cell* in the CPM is the set of all points in 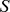 with the same cell index, 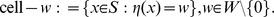 The value 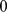 is assigned to a given node, if this node is not occupied by a cell but by medium. Each cell is of a certain *cell type*, which determines the migration and interaction properties of the cell, the set of all possible cell types being denoted by 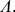 Denote by 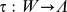 the map that assigns each cell its cell type. A cell with index 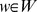 has volume

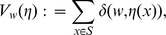
where the Kronecker symbol 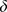 is defined by 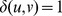 if 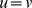 and 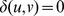 otherwise, and surface length



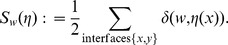



The sum in the last term is taken over all *interfaces* of a given configuration 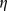, that are all pairs of lattice neighbors which do not belong to the same cell.

A cellular Potts model (CPM) is a time-discrete Markov chain with state space 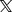, where the transition probabilities are specified with the help of a *Hamiltonian* or *energy*. The latter is a function 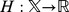 which often has a special structure. Usually it is the sum of several terms which are supposed to control single aspects of the cells' interdependence structure. Basically two terms are used in most CPM studies. First a *surface interaction term*

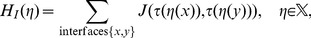
(1)is specified. Here 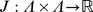, the matrix of so-called *surface energy coefficients*, is assumed to be symmetric. Second a *volume constraint*

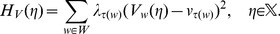
(2)is used. Here 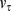, the target volume, and 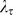, the strength of the volume constraint, are cell-type specific parameters, 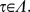 Dependent on the actual situation that shall be studied with the help of a CPM, further addends can be included, for instance a surface constraint [3]

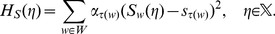
(3)Again 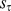, the target surface length, and 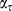, the strength of the surface constraint, are parameters, 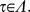 Thus, the typical structure of a CPM-Hamiltonian is
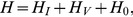
(4)where 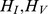 are given in (1) and (2) and 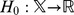 is a model-specific addend. Transitions from one configuration to another follow a special rule which is called *modified Metropolis algorithm.* First two additional parameters 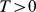, the so-called temperature, and 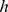, a transition threshold, are specified. Note that the transition threshold was set to 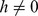 in the original model proposed by [2] but it turned out that in some applications 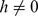 is a better choice [7], [22]. Then the following algorithm is performed:

1. Start with configuration 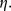

2. Pick a target site 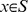 at random with uniform distribution.
3. Pick a neighbor 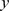 of 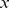 at random with uniform distribution among all lattice neighbors of 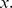

4. Calculate the energy gain, 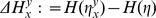, that is reached if the present configuration 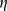 is replaced by the trial configuration 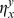. The latter is obtained from 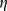 by copying the index 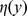 onto the node 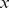, that is 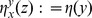 if 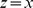 and 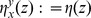 otherwise.
5. If the energy gain is below the transition threshold, that is if 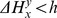, accept the trial configuration and put 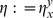; go to step 1. Otherwise, put 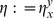 with chance 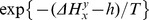 and keep 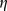 unchanged with chance 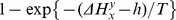.

Consequently, only such transitions are possible where the index of at most one lattice site is changed, resulting in a shift of the cell's center of mass. The new assignment to this lattice site is chosen from the cell indices of the neighboring lattice sites. These dynamics are interpreted to resemble membrane fluctuations, where one cell shrinks in volume by one lattice site and a neighboring cell increases in volume by occupying this site.

To complete the model, appropriate boundary conditions must be specified. If the influence of the boundary shall be neglected, periodic boundary conditions are used. This means that the space can be thought of as being mapped onto a torus. However, fixed boundary conditions, where the interaction between the cell surfaces and the confining environment is explicitly modeled, can be defined within this modeling framework, as well.

It will turn out, that most of the properties of the CPM that shall be discussed within this article do not depend on the specific structure of the Hamiltonian 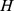. Therefore, it is assumed in the following that the Hamiltonian is a real function on 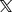 without stipulating a special structure such as (4). This approach has the additional advantage that boundary conditions can be included by adjusting the Hamiltonian accordingly.

#### Definition

Let 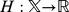 be a real function on 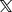 and suppose that 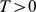. A *cellular Potts model* is a discrete-time Markov process with state space 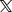 and with transitions following the modified Metropolis algorithm with respect to 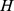 and 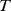.

The CPM model formalism has been used for several problem-specific extensions. In general, this is done by including additional terms into the Hamiltonian (4). For instance, elongated cell shapes can be modeled in a CPM by imposing a cell length constraint which renders the major axis of the ellipsoidal approximation of the cell's shape to be close to some prescribed target value [23]. Rod cell shapes with particular stiffness have been modeled using a compartmentalized cell concept, where each cell consists of a row of standard CPM cells [12]. In some cases, the kinetics of the original CPM is altered by directly modulating the transition rates that are calculated in step 4. of the modified Metropolis algorithm. Specific control terms that may depend on the configuration of the system but also on addition system parameters, like the position 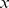 of the target site, the position 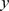 of the trial spin or the velocity increment of the affected cell, are added in step 3. of the modified Metropolis algorithm to the energy gain 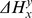 that is calculated from the Hamiltonian. Notice that these models cannot be represented within the classical model since the control terms cannot be derived from a Hamiltonian. Therefore these models with *kinetic extensions* will be referred to as *extended CPMs*. Examples comprise the explicit modeling of inertia by constraining the cell velocity increment [24] or the inclusion of chemotactic responses to some field 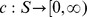 of signals into the model as in [3], [25].

Another extension of the CPM framework comprises *hybrid CPM models*. The standard CPM treats cells as internally structureless lattice domains. However, several studies have adapted the CPM to allow the modeling of subcellular structures, as well. The latter are derived from models of the intracellular biochemistry, typically modeled in terms of ordinary differential equations. In this way, the spatial configuration of the cells, their sizes, shapes, motility properties as well as the intercellular interaction can be coupled with cell-intrinsic processes. For instance, the effect of intracellular actin dynamics on membrane protrusions and retractions is modeled via a hybrid CPM in [21].

## Results

### Equilibria of the cellular Potts model

The standard Metropolis algorithm, see for instance ([1], §4.3), differs from the rules 0.-4. described in the previous section Steps 0. and 1. remain unchanged. In 2., the cell index 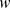 that is chosen to replace 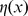 with some probability, is drawn uniformly from 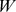 without considering the neighborhood of 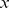. Then, in 3., a trial configuration 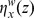 with


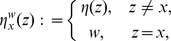


is used and the energy difference is calculated via 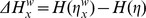. Afterwards an attempt is made to replace 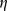 by 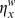 according to the decision rule in step 4.

The standard Metropolis algorithm is a Markov chain Monte Carlo method to explore the equilibrium model corresponding to the Hamiltonian 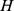. It samples typical configurations of the so-called *Gibbs measure* corresponding to 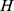. The latter is a measure 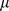 on 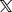 defined by
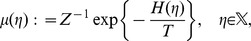
where 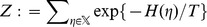 is the normalizing factor. This measure is a widely accepted model of statistical physics for the equilibrium behavior of particle systems at temperature 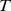 whose microscopic interdependence structure is described by 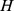. The transition rates 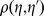 for transitions 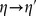 of the standard Metropolis algorithm satisfy the *detailed balance conditions* w.r.t. 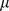, that is



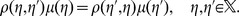
See ([1], §4.3) for details. Therefore, the Markov chain generated by the standard Metropolis algorithm has the Gibbs measure 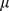 as a reversible measure. Since this chain is finite and irreducible, 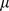 is its only invariant measure and the distribution at time 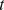 converges to 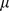 for 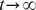. Thus, starting from an arbitrary initial configuration, the standard Metropolis algorithm produces realizations which are distributed approximately according to 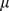 after a sufficiently long relaxation time.

The modified Metropolis algorithm does not satisfy the detailed balance conditions w.r.t. 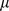. Indeed, it is easily verified by applying the results in ([26], Thm. 4.1) that the transitions in the CPM cannot satisfy detailed balance w.r.t. any measure neither related nor non-related to 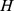. The main argument behind this statement is as follows. A cell in the CPM that covers only a single node has a positive chance to disappear during a transition, while the probability to reappear is zero. This behavior is a direct consequence of the modification in the Metropolis algorithm and is not present in the classical method. Therefore, the Markov chain defined by the CPM dynamics has several absorbing states, namely all those configurations that consist of only one cell that covers all nodes of the lattice. As it is shown rigorously in the Methods section, a CPM is eventually trapped in one of these absorbing states regardless of the special structure of its Hamiltonian. Its distribution 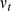 at time 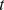 converges as 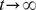 to a measure 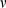 that is a convex combination of point measures 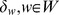, each of them concentrated on a constant configuration 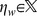 with 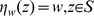. In detail, 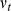 converges towards
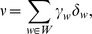
where 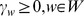, and 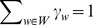. The weight 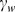 depends on the initial configuration 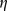 and equals the probability that the CPM started in 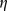 is absorbed by the constant configuration 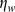. The values 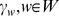, can be calculated explicitly, see the Theorem 1 in the Methods section. The time until absorption depends on the structure and parameter values of the Hamiltonian. Estimates of the time till absorption are provided in the Methods section.

The differences between standard and modified Metropolis algorithm become particularly blatant when 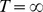 (or 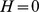). In this case, the CPM is a multi-type *voter model* [27], since in each transition the cell index of the target site is replaced by the cell index of a randomly chosen neighboring site. Consequently, the longtime behavior agrees with that of the voter model, where the cells consecutively die out. In contrast, the standard Metropolis algorithm decouples for 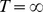, that means the evolution of each lattice site is independent from that of the other lattice sites and one observes a uniform distribution of spins in the long-time limit. Note that, for large temperatures, it was observed but not studied in detail in [2] that in a CPM ‘the pattern loses energy simply by eliminating cells’, which is a clear cue towards absorption.

Thus, it turns out that the modifications in the updating algorithm change the longtime behavior of the corresponding Markov chain dramatically in comparison with the standard, equilibrium model. Since detailed balance w.r.t. the Gibbs measure related to 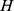 is broken and absorbing states are present, the long-time behavior is no longer controlled by the Hamiltonian 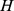. The modifications in the Metropolis algorithm, which could seem to be marginal, produce a qualitatively different behavior. In the long run, the evolution in the CPM is not directed towards the minimization of the energy 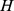 but the cells in the CPM consecutively die out.

### Impact of the Hamiltonian on the model dynamics at different time scales

It is pointed out in subsection Equilibria of the cellular Potts model that the modification of the Metropolis algorithm has a major impact on the dynamics. By relating the transition mechanism to the cell indices in the neighborhood of the target site, the impact of the Hamiltonian on the actual transition probabilities is reduced and even vanishes in the long-time limit. Still, it might be objected that the phenomenon of successive cell extinction in a CPM only marginally affects its behavior in parameter ranges that are of interest in the applications and that the above considerations are of theoretical value only. The main arguments in this direction are as follows. Firstly, a pragmatic ‘no-extinction’-rule for the CPM cells could be implemented. Secondly, it might be argued that the disappearance of CPM cells is so seldom that it can be neglected and that interesting and complex behavior is observed in the CPM before the process of cell extinction becomes manifest. The third objection could be that the role of the Hamiltonian 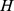 is purely technical to give the transition rates a bias and that the focus of interest in CPM studies is put neither on the long-time behavior nor on the minimization of the energy 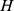.

However, there is a methodological problem with these arguments. A modeler controls the structure and the parameters of the Hamiltonian. With the help of the Hamiltonian, he implements his ideas about the underlying biological process into the model. The transition probabilities and thus the model kinetics, however, depend not only on this controlled term but also on a non-controlled voter-like part, stemming from the modification of the Metropolis dynamics, which depends on the geometric composition of a target spin's neighborhood. The latter part of the dynamics is sensible, for instance, to the topology of the underlying lattice, to the size of the chosen neighborhood structure and to the number of subcellular parts a CPM cell is divided into. These are technical parameters that have no mechanistic role for the biological process of interest. Thus, the CPM dynamics is characterized by a constant conflict of controlled behavior specified by the Hamiltonian and a non-controlled impairment.

The impact of the Hamiltonian, the controlled behavior, vanishes in favor of the non-controlled behavior for certain – long – time scales, as is discussed in subsection Equilibria of the cellular Potts model. To exploit the model in possibly well-behaved parameter regions, it is essential to address the following questions. What characterizes those regimes of a CPM, where the non-controlled, voter-like part of the transitions is marginal for the emergent behavior compared to the part of the transitions that is controlled by the Hamiltonian? To which extent does this regime depend on the structure and parameters of the Hamiltonian? To which extent does this regime depend on the dimension and topology of the underlying lattice and the grain size of the subcellular segmentation? What are the typical time scales that separate ‘good’, controlled behavior from ‘bad’, largely uncontrolled behavior? These theoretical questions have not been addressed in depth so far but need to be thoroughly discussed, if the results that are obtained from the analysis of a CPM are to be carried over into biological understanding.

Thus, coming back to the above objections, it can be argued that, firstly, a pragmatic ‘no-extinction’ rule for the CPM cells – as it is implemented in many applications, sometimes without explicit notice [28] – conceals the underlying conflict between controlled and uncontrolled behavior in the CPM. It has no mechanistic biological interpretation since it aims at a symptom that is of model-technical origin. Secondly, if the complex behavior observed in the CPM before the process of cell extinction becomes manifest shall be exploited for biological comprehension, it is necessary to validate the model appropriately. The interpretation of the model outcomes in biological terms is valuable in those cases where the extent of possible non-controlled influences is clearly assessed. This is particularly important, when quantitative predictions are to be derived. Thirdly, the focus of interest in CPM studies is predominantly in identifying the distinctive signature at the tissue level that emerges from specific intercellular interactions. It is characteristic for emergent phenomena, that the effect of the super-positioned microscopic interactions becomes not evident at the macroscopic spatial scale until a certain time – also measurable at a macroscopic scale – has elapsed. Therefore, numerical studies of the long-time behavior of CPMs and approximative descriptions of the their dynamics particularly at long, macroscopic time scales are indispensable.

Thus, the Hamiltonian 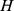 has a technical role by favoring those transitions which lower the energy. However, the dynamics is not driven by the Hamiltonian alone but there is a constant conflict with a non-controlled voter-like portion in the transition rates. This conflict becomes particularly blatant in the long-time behavior. The consequences of this conflict within other parameter regimes are not yet well-understood. The ambiguity in the model's dynamics constitutes a serious restraint for utilizing the CPM as a mechanistic model.

### Utility for spatio-temporal limit procedures

Typical properties of a spatially explicit dynamical model are often revealed by applying suitable spatio-temporal limit procedures. For instance, the ensemble 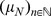 of Gibbs measures on increasing cubes 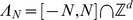 is studied in statistical physics. The cluster points of these measures are Gibbs measures on the infinite lattice 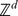. By studying the latter objects the phenomenon of phase transitions in the original model can be understood. In the case of the two-type voter model, the longtime behavior of spatially bounded systems is always trivial, that is the system is absorbed in one of the two constant configurations. However the extension of the voter model to infinite lattices shows a more complex behavior in dimensions 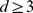 [27], [29], [30]. Exploiting the fact that spatially large but bounded systems are ‘close’ to infinite systems, the characteristics of the clustering process before absorption can be derived [31]. Another important tool of analysis is the derivation of a spatially and temporally continuous description of the considered process by sending the lattice spacing and the time unit to zero. The resulting model, which is often a partial differential equation, can be considered to be a description of the emergent macroscopic behavior that arises from the microscopic interaction [32], [33].

To carry out spatio-temporal limit procedures rigorously, it is necessary that the original Markov chain model on 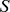 can be extended to the infinite, spatially unbounded lattice. Assuming, for simplicity, that S is a 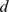-dimensional cubic lattice, an extension of the model to 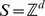 is required. The specification of transition probabilities is no longer sufficient for the description of the model, since the state space 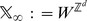 is innumerable then. This becomes apparent, for instance, if it is tried to perform the Metropolis algorithm on an infinite lattice. Actually, it is not self-evident that an extension of the model to spatially unbounded domains exists as a mathematically well-defined object. However, such an extension is straightforward, if the model can be interpreted as an interacting particle system (IPS) in the sense of Liggett [29]. This is the case, if the following two conditions are satisfied.

1. The original Markov chain model is temporally continuous.
2. The transitions are local.

Condition (1) is no serious restriction, since a time-continuous Markov chain can be constructed from a time-discrete Markov chain by a standard procedure. Indeed, let 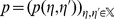 be the transition matrix of the original Markov chain model and define
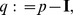
where 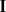 is the unit matrix on 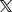. Then 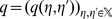 satisfies 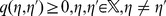
and 
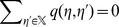
. Hence 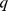 is a rate matrix which generates a time-continuous Markov chain. The evolution of this chain is very close to that of the original chain. The only difference is that the time to the next attempted transition is now exponentially distributed instead of constant.

Condition (2) is essential for defining a Markov process on 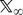. Transitions are local, if

(2-I) the configuration is changed only locally when a transition is performed;

(2-II) to calculate the transition rate, it is sufficient to know the configuration within a local neighborhood of the region that is to be changed.

A mathematically precise formulation of these conditions is given in the Methods section. In a CPM, the transition rates of the time-continuous algorithm satisfy (2-I) since only one site is changed in an (infinitesimal) transition. However, (2-II) is not satisfied, since the volume constraint 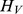 (2) is a non-local function. Indeed, to assess the impact of a cell-index change at location 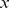 on 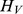, the volumes of the affected cells need to be known. These volumes can be determined only if the configuration on the whole lattice is examined. It is not enough to scan an appropriate neighborhood of 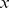. A detailed discussion of this issue can be found in the Methods section.

Thus, a CPM would be qualified for the application of spatio-temporal limit procedures, if it had a natural extension to spatially unbounded systems. However, the non-local nature of the transition rates blocks the integration of a CPM with Hamiltonian (4) into the model class of interacting particle systems in the sense of Liggett [29]. The methods that are available for CPM analysis so far comprise essentially numerical simulation studies, such as [7], [34], and heuristic approximations as in [35], [36], for instance. This reveals a present challenge when exploiting CPMs, since the extent of additional insight that can be gained by applying the model as well as the stringency of the conclusions within the model depend strongly on the capability and the rigor of the available analytical tools.

### Multiscale modeling within the CPM framework

CPMs are typically utilized to study the tissue scale properties that result from specific intercellular interactions. In extended CPM models such as [8], [10], [15], [21], [23], [25], [37], intracellular or molecular details are included additionally. Therefore, since multiple spatial and temporal scales are coupled into one description, CPMs are considered to be *multi-scale models*.

**Figure 2:**
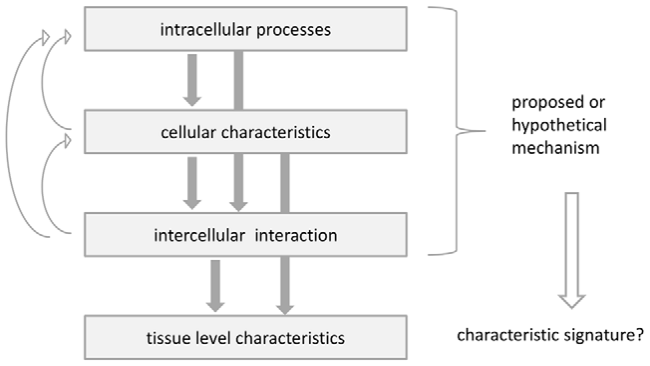
A mechanistic multiscale framework is characterized by the coupling of multiple spatial and temporal scales on the basis of abstracted rules. The assumed intercellular interaction may depend on an interplay with cellular characteristics and intercellular details. By determining the distinctive characteristics at the tissue level and their comparison with experimental observation, it can be tested wether a specific mechanism explains the behavior of an experimentally studied cell system.

There are two principal classes of such models that need to be distinguished. *Mechanistic models* evolve according to rules that have been abstracted from the underlying biological process. These rules represent a proposed or hypothetical mechanism concerning the intercellular interaction. The latter may depend on cellular characteristics and intracellular processes. The goal of developing a mechanistic model is essentially to provide a proof-of-principle for a proposed mechanism or to ‘verify’/falsify a hypothetical mechanism. This can be accomplished by determining – with the help of the model – the distinctive characteristics at the tissue level which emerge from the assumed intercellular interaction and their comparison with experimental observation. A scheme of this mechanistic multi-scale framework is depicted in Fig. 2. The main challenge of mechanistic models lies in accounting for the appropriateness of the model class by assessing the possible impact of simplifying model assumptions on the intended mechanism.

In contrast, a model of interacting cells is *phenomenological*, sometimes also called *empirical* or *data-based*, if it agrees with observed biological behavior in a statistical sense but its intrinsic mechanism of evolution is secondary. The models principal qualification for the studied question needs to be verified on an appropriate data basis. If adequate agreement is reached, phenomenological models can be exploited to explore theoretically the tissue scale outcome at conditions that resemble or complement the experimentally tested ones, thereby describing, inter- or even extrapolating existing biological data. The biological experimentalist can thus be guided towards the most interesting behavior. The main challenge of phenomenological models lies in validating the model appropriately, that is to identify and match particularly those characteristics that are critical for accordant model behavior.

Thus, mechanistic and phenomenological models are used for different purposes. Sufficiently validated phenomenological models can be utilized to summarize and visualize data, to make predictions and as heuristics for designing experiments. Mechanistic models with well-founded model assumptions can also provide predictions of the system's behavior but the focus is put rather on an satisfactory explanation of the observed phenomena. Notice that phenomenological models might be constructed partly by mechanistic considerations. However, if the exploited model behavior is not robust with respect to additional, purely technical model assumptions or a full parameter variation within the biologically explained constraints, its explanatory power becomes ambiguous.

**Figure 3:**
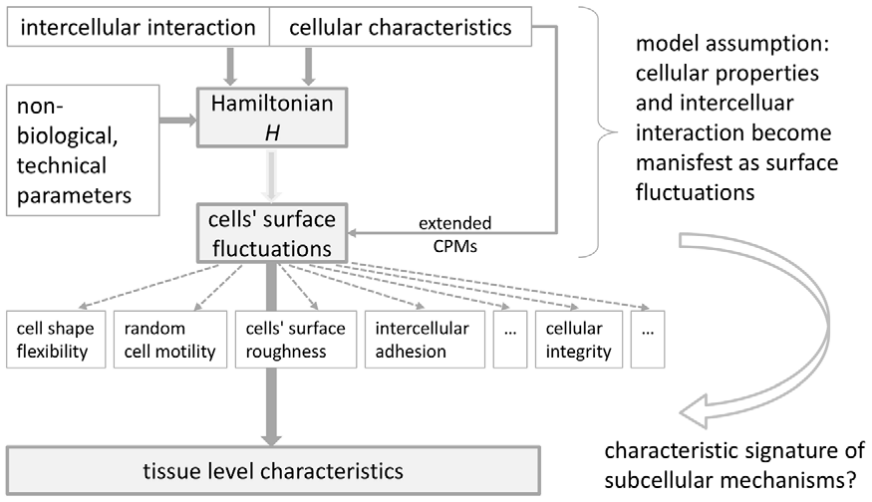
Cell surface fluctuations are the central device in the realization of the multiscale concept in CPMs. Both the rules of intercellular interaction and the considered cellular characteristics are eventually coded, via the Hamiltonian or directly for extended models, into an expression that regulates the intensity of CPM-cells' surface fluctuations. Additional technical parameters are integrated into the Hamiltonian to be able to suppress phenomenologically unrealistic behavior. The actual impact of the Hamiltonian on the intensity of CPM cells' surface fluctuations is attenuated by a voter-like portion in the transition rates. The surface fluctuations drive simultaneously the actual behavior of a CPM at the cellular scale, the specifics of intercellular interaction and the emerging behavior at the tissue scale. Single aspects of the cellular properties in the model, for instance the cell shape flexibility, the magnitude of random cell displacements or the cells' surface roughness, and of the intercellular interaction, like the strength of intercellular adhesion, cannot be controlled individually but are interlinked with each other. Likewise, purely model-technical control parameters such as the cellular integrity, that is the property of CPM cells to span over connected, essentially convex lattice domains, are coupled indirectly with biologically interpretable cellular and intercellular properties. The emerging tissue scale behavior is solely rooted in the specified characteristics of the CPM cells' surface fluctuations and not linked directly to cellular and intracellular specifics.

The mechanistic construction of a CPM is displayed schematically in Fig. 3. The central device in a CPM are the CPM cells' surface fluctuations. They are mainly regulated by the Hamiltonian 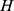, but the actual control of the Hamiltonian over the intensity of the surface fluctuations is attenuated by a voter-like portion in the transition rates, as is discussed in the subsection on the Impact of the Hamiltonian. The Hamiltonian is a sum of terms, typically at least the surface interaction term 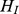 and the volume constraint 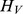, see (1) and (2), respectively, which are assumed to reflect simultaneously the effect of the intercellular interaction and of the cellular characteristics. Supplementary terms, such as the surface constraint 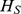, see (3), are integrated into the Hamiltonian to further enforce phenomenologically realistic behavior. All these terms are, however, not derived from a mechanistic assumption about the behavior and interaction of the subcellular parts that are resembled by the single nodes of a CPM cell. Instead, these terms describe heuristically the effect of all determinants – from the subcellular to the intercellular scale – which are assumed to become ‘somehow’ manifest as cell surface fluctuations. Thus, the parameters of a CPM Hamiltonian can be dived into (i) directly biologically interpretable or measurable parameters, like the cells' target volumes 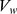, (ii) effective parameters that subsume various intercellular processes and cellular details, such as the surface interaction strengths 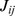, and (iii) merely technical parameters with ambiguous biological interpretation, like the parameters 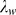 and 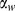, 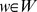, which determine the impact of the volume and surface constraints. The temperature 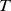, which weights the overall impact of the Hamiltonian on the dynamics, is also a purely technical parameter. In the model, the parameter 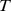 controls the strength of interaction between neighboring lattice nodes. The higher 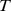, the less dependent they evolve. Thus, it affects at the same time the subcellular cohesion, the intercellular interaction and the degree of control that is exerted via the Hamiltonian onto the surface fluctuations. There is a continuing obscurity concerning the interpretation that can be given to this parameter [2], [3], [11], [15]. It seems that it remained from the physical origin of the model. Notice, that the parameter 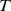 of the CPM can be omitted by a scaling 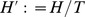.

The surface fluctuations drive simultaneously the actual behavior of a CPM at the cellular scale, the specifics of intercellular interaction and the emerging behavior at the tissue scale. Single aspects of the cellular properties in the model, for instance the cell shape flexibility, the magnitude of random cell displacements or the emerging cells' surface roughness, and of the intercellular interaction, like the strength of intercellular adhesion, cannot be controlled individually but are interlinked with each other. Likewise, purely model-technical control parameters such as the cellular integrity, that is the property of CPM cells to span over connected, essentially convex lattice domains, are coupled indirectly with biologically interpretable cellular and intercellular properties. The emerging tissue scale behavior is solely rooted in the specified characteristics of the CPM cells' surface fluctuations and not directly linked to cellular and intracellular specifics.

If a CPM shall be utilized as an explanatory mechanistic model, the central role of the surface fluctuations constitute a handicap for a thorough understanding and interpretation of the model outcome. This is because the single aspects of cellular behavior in a CPM cannot be assessed individually by the modeler but are coupled to each other in an indirect fashion. An observed signature at the tissue scale might be traced down to the underlying intensity of surface fluctuations. However, it is hardly possible to identify and separate the effect of single components in the model's interplay of intercellular interaction, cellular characteristics and even intracellular or molecular details that is apparent as cell surface fluctuations.

Thus, a CPM's spatial resolution below the cellular level, which is the basis for modeling deformable cells, and the central role of the surface fluctuations entail substantial drawbacks for its mechanistic construction and, consequently, the explanatory power of the model. If flexible, adaptive and non-isotropic cell shapes or a variability in the cells' sizes are assumed to essentially affect the intercellular interaction, a CPM is one of a few existing models that can be applied to show that a proposed mechanism ‘somehow’ produces the observed behavior. Disagreement with the biological data, however, could mean both: the incapability of the model to correctly capture the postulated mechanism or the inappropriateness of the hypothetical mechanism for the biological system at hand. Therefore, mechanistic conclusions drawn from CPM models are only limitedly reliable. A validation of the results with the help of alternative models which operate at differing levels of complexity and thus represent different modeling compromises is worthwhile. If cell sizes and shapes are of minor importance for the interaction, more coarse-grained individual-based approaches such as interacting particle systems, e.g. [29], [38]–[40] may lead to mechanistically better understood and analytically easier tractable models.

If a CPM shall be exploited as a phenomenological model, it is necessary to empirically match the kinetic properties of the model with the respective data from the relevant biological experiments. The benefit of this approach depends on the availability of representative data for parameter estimation and model validation as well as on theoretical knowledge about the fundamental dynamical properties of CPMs. Although blatantly unrealistic cell behavior is prevented by modifying the standard Metropolis algorithm, it is not self-evident that the kinetic properties of a given CPM with Hamiltonian 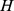 really resemble the dynamical behavior of the considered experimental system. The challenge consists in (I) identifying the key determinants that have to be empirically matched in model and experiment to maximize the descriptive power of the model and in (II) defining suitable measures of the descriptive and predictive performance with respect to specific traits. To progress in this direction, it is necessary to have a substantial knowledge of the model's principal behavior in the respective parameter regions. So far, there are a few studies that analyze the kinetic properties of CPM's and compare them to corresponding characteristics found in experimental data. For example, [34] studied numerically the relaxation kinetics for clustering in the original CPM introduced by [2] for cell sorting, thus providing a reference framework for the model's comparison with experimental data. Other examples include [12] who assess the biological plausibility of single cell dynamics before they turn towards their actual focus of study, the collective behavior that results from alignment rules based on elongated cell shapes, and [41] who provide empirical guidelines about how to tune a CPM in order to optimize its behavior with respect to certain biophysical characteristics.Notice that the necessity to empirically match simulations and experiments has already been recognized in [42]: ‘A first step towards quantitative cell-based modeling is to ensure the cell behavior modeled by the CPM matches experiments exactly.’ Here, it shall be emphasized that tuning the parameters of a model such that it empirically matches the observations with respect to certain key characteristics – regardless of the qualitative or quantitative nature of the conformance measures – implies that the model is utilized predominantly as a phenomenological model. Those models describe or visualize rather than explain experimentally observed behavior.

To describe a given experimental situation by a CPM, it is necessary that there is a protocol of how to adjust the model parameters in such a way, that the ‘in silico’ condition is comparable with the actual preparation of the biological system. Since the CPM parameters interfere with each other in a complex way, a simultaneous parameter fitting is often applied. At present there is no standard algorithm for the model adjustment. Instead, it is a very intricate task that requires much intuition and skill by the user [28].

Vice versa, if the behavior at conditions that have not yet been explored experimentally shall be predicted, it is essential that a concrete ‘in-silico’ setting can be translated into a biologically condition that is defined by the states of certain experimentally manageable quantities. However, several CPM parameters, in particular those which subsume various intercellular and cellular details into unspecific effects becoming manifest as surface fluctuations, like the surface energy coefficients 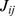, are hard to match with biological traits. Varying 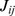, for instance, affects the details of intercellular interaction and, simultaneously, the cell motility, the magnitude of surface fluctuations and the smoothness of the cell surface: ‘more cohesive cells [in a CPM] have more crumbled surfaces, larger membrane fluctuations and diffuse further than less cohesive cells’ [3]. Even the actual cell size in the CPM is affected: ‘... cells with a higher surface energy (but the same target volume) overall are smaller ...’ [8]. There are a number of observed tissue scale characteristics in the CPM that have a direct biological interpretation, such as the average magnitude of cell center displacements within the cell population, the actual average magnitude of surface fluctuations, the apparent smoothness of cell surfaces, or the observed distribution of cell sizes. However, the attribution of these emergent characteristics to manageable quantities at the cellular and intercellular scale, such as the strength of intercellular adhesion between two cells, the degree of the intrinsic motility of an individual cell, or even the expression profiles of certain molecules at the cell surfaces, is sometimes rather vague.

Thus, the central role of the surface fluctuations for the CPM dynamics entails substantial drawbacks for its exploitation as a phenomenological model of collective cell behavior. If the key characteristics of the studied biological system that have to be matched by the model system include flexible, adaptive and non-isotropic cell shapes or a considerable variability in the cells' sizes, a CPM is one of a few existing models that can be applied.

The exploitation of a CPM as a phenomenological model is also reasonable if the morphometric composition and the spatial arrangement of the CPM cells and their dynamic reorganizations solely constitute the spatial structure for the study of coupled intracellular and molecular processes. In this case, the focus of interest is put on analyzing the patterns and structures that emerge from the interaction of these processes, for instance by modeling them as coupled ODE systems. If the underlying spatial structure shall not be static but temporarily varying or even be slightly adaptive to the modeled intracellular occurrences, a CPM can provide such a spatial framework. A CPM that is empirically adjusted to match the key determinants of the morphometric cell composition and the spatial cell arrangement in the given experimentally assay, can be utilized then as the spatial basis for an additional, mechanistic modeling stacked on top on it.

## Discussion

CPMs are typically applied if the tissue scale properties that emerge from specific intercellular interactions shall be described, predicted or explained. The model class originates in statistical physics, where Markov chain Monte Carlo methods are utilized to study the behavior of many interacting particles at equilibrium. The model's adaption to the requirements of modeling morphogenetic processes involves a paradigm shift from equilibrium to non-equilibrium, kinetic modeling. The temporal evolution in the model – which before has been an auxiliary tool to drive the system towards the equilibrium state of interest – turns out to be the core of the transition mechanism in a CPM. Correction terms in the CPM Hamiltonian and additional modifications in the original transition mechanism help to eliminate biologically unrealistic behavior. The modifications in the transition mechanism dramatically alter the long-time behavior of a CPM compared to its counterpart in statistical physics. In the long-run, the temporal evolution of a CPM is not directed towards the minimization of the Hamiltonian or energy but instead the CPM cells consecutively die out. The correction terms in the Hamiltonian render the transition mechanism to be non-local, thus hindering the application of powerful analysis methods from statistical physics such as spatio-temporal limit procedures. Thus, the descent from a well-studied model class in physics can hardly be exploited for CPMs.

To assess whether CPMs constitute good dynamical models for multi-scale problems in morphogenesis, it is helpful to distinguish between the intended purpose of modeling: mechanistic modeling can be applied to explain an observed phenomenon, while phenomenological modeling rather describes biological observations. In both cases, the mechanistic construction of the CPM dynamics, where the intensity of the surface fluctuations is the central device that subsumes the effect of all cellular and intercellular details, entails substantial limitations for a CPM's exploitation in the respective direction. If considerable variability in the cell sizes and shapes or flexible cellular neighborhood relations are supposed to essentially determine the intercellular interaction, the CPM framework is one of a few model classes that can be utilized. The application of CPMs is also reasonable, if a non-static, dynamically changing spatial structure shall be simulated that forms the cellular basis for interacting intercellular and molecular processes. Then the focus is put on the patterns and structures that emerge from the interaction of these processes and the tissue rearrangement described by the CPM is of minor importance, utilized rather to represent the fluctuations in the spatial composition of the cell population. In those cases, however, where essentially isotropic, non-polarized cells of uniform size are considered, it is worthwhile to validate the results by comparing them to the outcomes of more coarse-grained modeling approaches, like Cellular Automata or Interacting Particle Systems, that are mechanistically better understood and analytically more accessible although they may look visually less appealing.

In most cases where a CPM is used, an important biological problem is addressed which is characterized by an interplay of several factors from different scales, acting at the intracellular, the intercellular and the tissue level. The developed CPM usually incorporates much detail and substantiates deep biological insight. Computer simulations can be an important tool for a deeper understanding. However, already the original system, which underlies all more elaborate CPMs, is still poorly understood in its theoretical and mathematical properties. The methods that are available for its analysis so far comprise essentially numerical studies and heuristic approximations. Since the stringency of the conclusion that can be gained by applying a model depends substantially on the capability and rigor of the available analytical tools, this presents a considerable challenge. The more details from the cellular, intracellular and possibly intracellular scale are included into the model the more pronounced are the challenges which are encountered when adjusting the model specifics to the biological situation at hand or when analyzing the model outcomes theoretically. To value the contribution of a CPM to the understanding of an underlying biological problem, it is essential that the theoretical characteristics of the model class are well-understood. Artifacts and non-robustness of the model behavior deserve particular attention, since laying them open helps to define the good of the model. This study provides a starting point for such work. It also constitutes a theoretical basis for developing assistance in constructing and choosing expedient model parameters and to give practical advice for cellular Potts implementations. Quantitative estimates for choosing the parameter values such that a CPM behaves as intended within certain time scales depend largely on the specific model that shall be applied. While the formulas to calculate the time to extinction for a given CPM are derived explicitly here, the development of further quantitative support for the CPM construction, the appropriate parameter choices and the determination of reasonable time scales for conclusive simulations is left to future studies.

Further theoretical analysis of the CPM class is worthwhile and shall be encouraged by this work. In particular, the study of highly simplified models may lead to mathematically well-founded assessments of the principal behavior of CPMs under various conditions concerning the temporal scales and the specific structure of the Hamiltonian as well as the parameter regimes. Besides this, the simultaneous representation and theoretical as well as empirical analysis of the same biological mechanism by various models which differ in their spatial resolution and particular model structure can help to distinguish the factors that are robustly described from the effects that must be attributed to the model's peculiarities. In this respect, the comparison between CPMs and non-lattice models, such as the Vertex model, or between CPM and cellular automata or interacting particle systems seems to be most promising.

## Methods

### Absorption for cellular Potts models

First, it shall be shown that any cellular Potts model as defined in subsection Mathematical model description is eventually absorbed by a constant configuration. Recall that 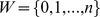 and 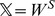, where 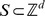 is a finite set. For 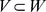, let be 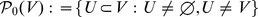 the set of non-empty proper subsets of 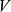. Define further
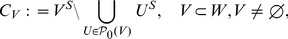
(5)the set of all configurations where exactly the cells with cell indices from 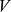 are present. Notice that, for 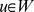, the set 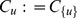 contains only the constant-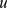 configuration 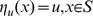.

Since the probability for the next transition in a given CPM is determined solely by the present state but not the past ones, the temporal evolution of a CPM is a Markov chain ([43], Def.2.1.1). The behavior of the latter is completely characterized by the transition matrix 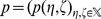, where 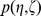 is the probability of a transition 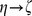 by one step of the modified Metropolis algorithm, 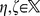.

In the following, the assertion that any CPM is eventually absorbed by one of the constant configurations is derived from considerations about the structure of the transition matrix. The findings presented here are based on results in the theory of finite Markov chains. See, for instance, ([44], §4-§5) or [43] for more detail.

#### Proposition 1

1. The sets 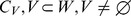, are the *communication classes* associated with the transition matrix 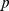.

2. For 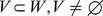, the class 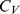 is *closed* if and only if 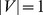.

3. The elements of
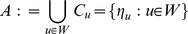
are the *absorbing states* associated to 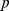 while the states that belong to 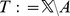 are the *transient states*.

4. If the configurations in 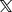 are arranged appropriately, the transition matrix has the form
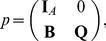
(6)where 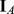 is the unit matrix on 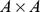, 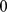 is the null-matrix on 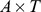, 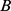 is a non-negative, non-vanishing matrix on 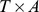 and 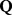 is a substochastic matrix on 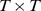.

Notice that a matrix 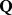 is *substochastic* if it has non-negative entries with row sums less or equal than one but strictly less than one for at least one row.

#### Proof

(1) Given two configurations 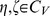, it is possible to reach 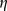 from 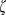 by performing a finite number of transitions each with positive transition probability, and vice versa. Thus, the elements of 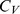 are communicating ([43], Def.2.4.1). If a configuration 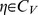 is given, each set 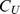 with 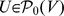 can be reached by performing a finite number of transitions each with positive transition probability, but the sets 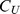 where 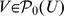 cannot be reached in this way. Therefore, the sets 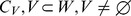, are the communication classes associated with the transition matrix 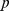 ([43], §2.4.1).

(2) One observes that


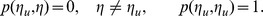


Therefore, the states 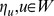, are absorbing and the singletons 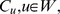 are closed communication classes ([43], Def.2.4.2). Since the class 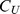 is accessible from the class 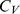, if 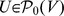 and since 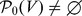 for 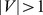, it can be concluded that for 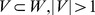, there exists an 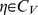 such that



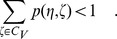
Therefore, the classes 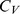 are not closed if 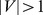.

(3) Any state of a Markov chain is either recurrent or transient, compare ([43], §3.1.1). Both properties are class properties ([43], Thm.3.1.2), that means either all elements of a communication class are recurrent or all class members are transient. A recurrent communication class is closed ([43], §3.1.3). Thus, one finds that
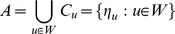
is the set of absorbing states associated to 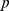, and 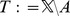 are the transient states.

(4) It follows from (3) that the transition matrix 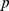 has the structure described in (4) if the configurations in 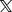 are arranged in such a way that the absorbing configurations from 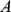 are followed by the transient configurations from 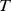.

Next, asymptotic properties of the matrices 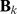 and 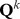 are derived, where 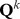 is the 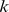-th matrix power of the matrix 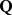 and 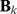 is defined by
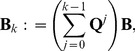
(7)both 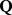 and 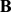 given by (6). This is motivated by the fact that the long-time behavior of a Markov chain with transition probability 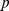 is completely determined by the 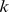-th matrix power 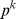 of the transition matrix 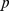 for sufficiently large 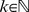. Indeed, if 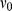 is the initial distribution, then



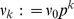
is the distribution at time 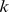 ([43], §3). Notice that
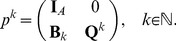
(8)


#### Proposition 2

(1) For any 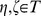, it holds that
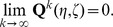



(2)
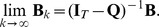



#### Proof

(1) ([44], Prop. 5.1(i)).

(2) Define 
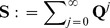
. Since
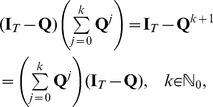
one obtains by letting 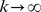 that



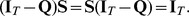
Thus, 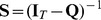. The assertion (2) follows now from (7) by letting 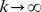. Indeed, one finds that



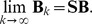



Now the results about eventual absorption and the probabilities of absorption by a particular configuration can be stated. Suppose that the Markov chain with initial distribution 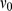 and transition matrix 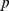 is denoted by 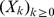 and the underlying probability space is denoted by 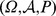. *Absorption* is the event that the Markov chain reaches one of the absorbing states within finite time, that is
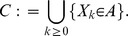



The event of being absorbed by a particular constant-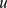 configuration 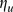 is defined by
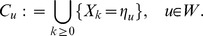



Denote by 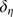 the Dirac or point measure in 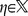.

#### Theorem 1

(1) For any initial distribution, absorption occurs almost surely, that is
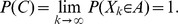



(2) If the Markov chain 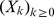 is started in the configuration 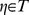, then the probability of absorption by the constant configuration 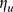 is given by
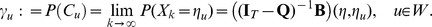



(3) The set of stationary distributions of a CPM is given by
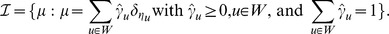



#### Proof

(1) One finds that the event 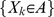 implies the event 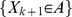, since the Markov chain cannot escape from an absorbing state once it was captured there. Consequently,
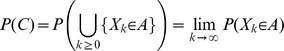
by the continuity of the measure 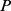.

For the distribution 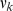 of the Markov chain at time 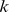, it holds that 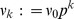, where 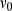 is the initial distribution and 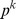 is the 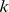-th matrix power of the transition matrix 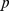. Since
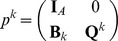
with 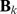 defined by (7), one obtains
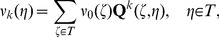
(9)and
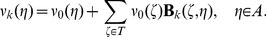
(10)Thus on can conclude



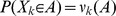


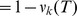


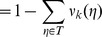


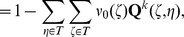
where the last equality follows from (9). By Proposition 2 (1), each addend converges to zero for 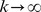. Taking into account that the sum consists only of a finite number of addends, the assertion is proven.

(2) Fix 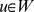. The event 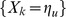 implies the event 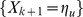, since the Markov chain cannot escape from an absorbing state 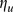 once it was captured there. Thus 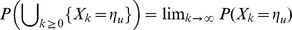 by the continuity of the measure 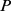. Since 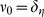, one obtains from (10) and Proposition 2 (2) that
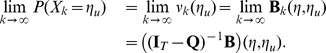



(3) Since the Markov chain is almost sure captured by one of the absorbing states 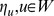, the set of stationary distributions is the convex hull of the point measures 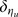 concentrated on the constant-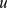 configurations 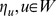.

### Time till absorption for cellular Potts models

A CPM's development towards absorption proceeds from an initial state where all cell indices of 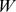 are present via the consecutive disappearance of single cell indices until the final absorbing state is reached. Therefore, the time of absorption can be estimated if the time until the first disappearance of a cell index, that is the time of the Markov chain exit time from the set 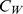, can be estimated. Let the random variable 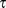 denote this time of exit from the set 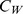,
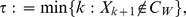
where 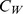 is given by (5). Thus, 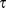 is the time where the first CPM cell dies out.

To state the results about the distribution of 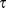, some additional notation is necessary. Notice that the transition matrix 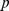 has the structure
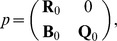
where 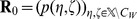, 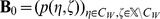 and 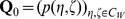, if the rows and columns of 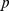 are arranged appropriately. Consequently, it holds that



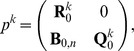
where 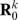 and 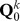 are the 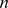-th matrix power of 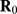 and 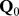, respectively, and 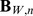 is some non-negative non-vanishing matrix composed from 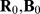 and 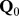. Further, 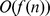 shall represent a function of 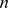 such that there exist 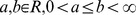, with 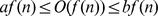 for all sufficiently large 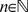.

#### Proposition 3

There exists a real eigenvalue 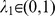 of 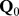 such that 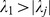 for any other eigenvalue 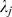 of 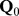. Moreover, the left eigenvector 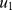 and the right eigenvector 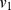 associated with 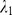 can be chosen positive and such that 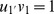, where 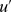 denotes the transpose of a vector 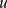. Suppose that 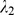 is an eigenvalue of 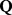 with multiplicity 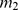 such that 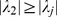 for all other eigenvalues which are different from 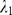 and 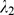. Then it holds that
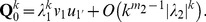
(11)


#### Proof

The matrix 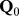 is substochastic and *primitive*. The latter property means that 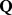 is both irreducible and aperiodic ([43], Def.6.1.2). Therefore, the Perron-Frobenius Theorem can be applied ([43], Thm.6.1.1), which proves the assertion.

A direct consequence is the following theorem.

#### Theorem 2

The distribution of 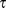 is approximately geometric with parameter 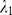, that is
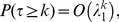
(12)where 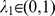 is the eigenvalue of 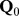 which is the largest in absolute value.

#### Proof

Define 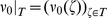 and 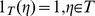. The distribution of 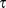 is computed by
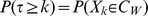


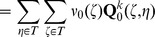


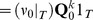


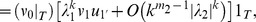


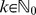
, where the latter equality follows from (11). Since 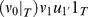 is a constant independent of 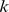 which does not vanish, the assertion is proven.

Thus, the path towards absorption is as follows. If a CPM started on 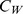 leaves this set, it runs next into one the sets 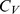, 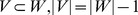. Almost surely, this happens within a finite number of time steps. The distribution of the exit time is approximately geometric. Once the chain has entered a set 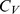 with 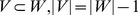, it cannot go back to 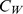. It stays in 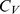 until the next cell dies out. In this way it successively visits classes 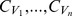 where 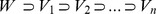. The differences between two consecutive classes are singletons. The set 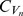 is absorbing, since it consists only of one constant configuration.

The time until absorption can therefore be estimated by consecutive application of Theorem 2. Notice that the future evolution of a CPM which has already entered a class 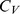 with 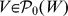 is the same as that of a CPM restricted to 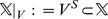. To restrict a CPM to the set 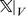, consider the restriction 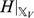 of the original Hamiltonian and perform the modified Metropolis algorithm on 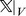. It turns out that the associated transition probabilities are described by 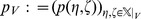. Consequently, the time of exit from 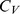 is again approximately geometrically distributed and the parameter of this distribution can be obtained as the leading eigenvalue of 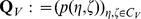 by applying the above arguments to 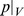.

### Locality of transition rates

The locality conditions (2-I) and (2-II) stated above represent a slightly informal interpretation of the finite range conditions for interacting particle systems (IPS) as defined in ([29], Def.I.4.17). Notice that IPS are continuous-time models while CPM evolve in discrete time steps. However, as explained in the section Utility for spatio-temporal limit procedures, a continuous-time Markov chain can be constructed from a temporally discrete chain by choosing the rate matrix 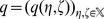 according to 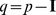. The matrix entries of 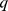 and 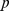 differ only in their diagonal entries. The diagonal entries, however, are determined by the off-diagonal elements, since, for stochastic and rate matrices, the row sums are equal to one and zero, respectively. This implies that the locality conditions, which are stated precisely in the following, can be examined on the basis of the transition matrix 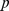 as well as on the basis of the corresponding rate matrix 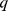.

Conditions (2-I') and (2-I'') as stated below are exactly the finite range conditions for IPS ([29], Def.I.4.17), however, they are adapted to the notation of this paper. Notice that locality conditions are important for ensuring the existence of a process on spatially unbounded lattices. Therefore, when considering a mechanism on a finite lattice which shall be extended to an infinite lattice, it is essential that the constants 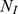 and 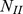 in (2-I') and (2-I''), respectively, are independent from the lattice size. In the following, Condition (2-I) and (2-II) shall be formalized. For this, define the diameter of a set by 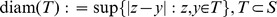, with 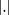 denoting the metric on 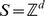 which is induced by the Euclidian norm on 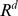. The set of points where two configurations 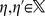 differ is given by . Further, denote by 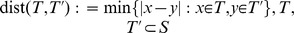 the distance of to sets. Then the precise locality conditions are as follows.

(2-I') There is a 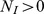 such that 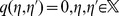, unless 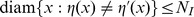.

(2-II') There is a 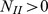 such that 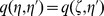 for all 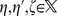 with 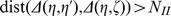.

#### Proposition 4

(1) A CPM mechanism satisfies condition (2-I').

(2) A CPM with volume constraint does not satisfy condition (2-II')

#### Proof

(1) The transition rates of the time-continuous CPM algorithm satisfy (2-I') since only one site is changed in an transition. Indeed, 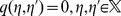, unless 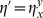 for suitable 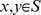 with 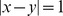. Thus 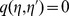 for 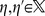 with 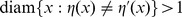.

(2) The rate for a transition 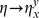 is a function of 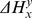,
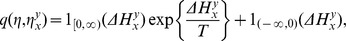
where 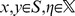. Thus the locality properties of 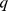 depend on the properties of 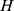. The typical structure of a CPM-Hamiltonian as given in (4) includes a volume constraint 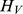 with



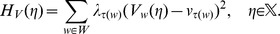



The difference 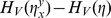 must be calculated to determine the transition rate for a cell-index change at location 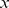. Due to the quadratic term, this difference depends explicitly on the volumes 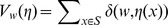 of the affected cells 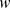 and not solely on the volumes' increase or decrease. However, to determine the volume of a cell in a CPM, it is not enough to scan an appropriate neighborhood of 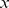. Therefore, the constant 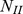 in condition (2-II') would depend on the lattice size, which means that (2-II') is not satisfied.

Two remarks are in order. First, it is easy to see by the above arguments, that any mechanism where the transition probabilities can be determined only if the configuration on the whole lattice is examined. This applies, in particular, to a surface constraint, but also to some proliferation or shape control mechanisms of CPMs. Second, the rates would be local, if the Hamiltonian 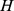 were constructed solely from a *finite range potential*. The latter is a family 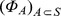 of functions
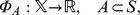
which satisfy for each 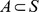


(I) 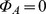 if 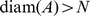 and

(II) 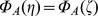 for all 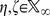 with 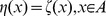.

Notice that the constant must be independent of the lattice size, if a potential on a finite lattice is studied. Given such a potential, a Hamiltonian can be constructed via
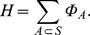



The Hamiltonians that are used in statistical physics are usually constructed from finite range potentials. Notice that the surface interaction term 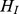 of a CPM can be derived from the finite range potential
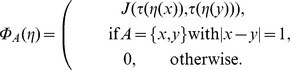



However, as soon as there is a non-local function like the volume constraint added, the CPM does not satisfy (2-II'). Actually, the locality condition on both the transition rates of an IPS as well as the finite range condition for the potential can be slightly relaxed ([19], Thm.I.3.9). Nevertheless the CPM does not fall into this model class.
